# The expression of renal Epstein-Barr virus markers in patients with lupus nephritis

**DOI:** 10.3892/etm.2014.1578

**Published:** 2014-02-24

**Authors:** XIAO-XIA YU, CUI-WEI YAO, JING-LI TAO, CHEN YANG, MIAN-NA LUO, SHANG-MEI LI, HUA-FENG LIU

**Affiliations:** Institute of Nephrology, Guangdong Medical College, Zhanjiang, Guangdong 524001, P.R. China

**Keywords:** autoantibodies, Epstein-Barr virus, lupus nephritis, renal tissues

## Abstract

The aim of this study was to investigate the role of renal Epstein-Barr virus (EBV) infection in the pathogenesis of lupus nephritis (LN). A total of 58 renal tissue samples from patients with LN, seven normal renal tissue samples from patients with non-glomerular hematuria and 37 renal tissue samples from patients with minimal change nephropathy were collected. The expression of EBV-latent membrane protein-1 (EBV-LMP1) and EBV-encoded RNA 1 (EBER-1) in the renal tissue was examined by immunohistochemistry (IHC) and *in situ* hybridization (ISH), respectively. The sera levels of anti-nuclear antibody as well as antibodies to extractable nuclear antigen in patients with LN were also measured. An equivalence test showed that the results from the IHC and the ISH analyses had strong agreement. The positive rates of renal EBER-1 and EBV-LMP1 in the LN patients were significantly higher than those of the normal and minimal change nephropathy patients (P<0.001), while no significant difference was identified between those of the normal and minimal change nephropathy groups (P>0.05). The positive rates of EBV-LMP1 and EBER-1 in the renal tissues of patients with LN were not determined to be significantly different between the relapse (immunosuppressant-treated) and initial onset (non-treated) patients, between the patients with and without concurrent infection, and among the patients with different age ranges (P>0.05). The proportion of LN patients positive for anti-Sm antibody was significantly higher in the renal EBV-positive group than in the EBV-negative group (P<0.05), while the proportions of LN patients positive for the other autoantibodies that were examined were not identified to be significantly different between these two groups (P>0.05). The present study shows that renal EBV infection may contribute to the pathogenesis of LN by inducing anti-Sm antibody production.

## Introduction

Systemic lupus erythematosus (SLE) is a multisystem autoimmune disease. The etiology and pathogenesis of SLE are possibly multifactorial; however, the mechanism of pathogenesis has not been fully elucidated. Genetic susceptibility and estrogen, as well as environmental triggers including viral infection, ultraviolet light exposure and drug use, may be involved in the immune dysfunction of SLE (1), among which viral infection has attracted the majority of attention (2–4) as it induces the production of antibodies (5). Since Epstein-Barr virus (EBV) was first reported by Evans *et al* (6) in 1971, the relevance of EBV infection in SLE has been continuously investigated. Thus far, the majority of the evidence suggesting EBV infection is involved in the pathogenesis of SLE has been obtained from viral antigens, the EBV genome or serological detection in the peripheral circulation of patients with SLE (7–13). The kidney is the most commonly involved organ in patients with SLE, which is subsequently named lupus nephritis (LN). To the best of our knowledge, whether renal EBV infection is involved in the pathogenesis of LN has not been reported. In the present study, the renal expression of gene and protein markers of EBV in patients with LN were detected.

## Materials and methods

All study methods were approved by the Ethics Committee of The Affiliated Hospital of Guangdong Medical College (Zhanjiang, China). Written consent of participation was signed by every subject enrolled in the study.

### Clinical data

In total, 58 renal tissue samples from patients with LN, seven normal renal tissue samples from patients with non-glomerular hematuria and 37 renal tissue samples from patients with minimal change nephropathy were collected by the Institute of Nephrology, Guangdong Medical College (Zhanjiang, China). All 58 patients with LN met the diagnostic criteria for SLE published by the American College of Rheumatology in 1997 (14) and manifested renal involvement, which was confirmed by clinical proteinuria and/or renal failure. Of those 58 patients, 52 were female and six were male, with a mean age of 27.5±1.0 years (range, 10–56 years). The duration of disease was between seven days and three years. The SLE disease activity index of the 58 patients was >10. All seven normal renal tissue samples were collected from patients with persistent unexplained hematuria, and it was identified by renal biopsy that the hematuria was of non-glomerular origin. Serum were also collected at the time of biopsy from patients with LN for autoantibody determination.

The patients with LN were divided into an initial onset group for those who had never received any immunosuppressants and a relapse group for those who had received immunosuppressant treatment. The LN patients were also divided into a non-infection group and a concurrent infection group for those who had suffered from respiratory infection, gastrointestinal infection, urinary tract infection, skin infection or other type of infection within 3 months prior to renal biopsy.

### Detection of EBV-latent membrane protein-1 (EBV-LMP1) expression using immunohistochemistry (IHC)

Briefly, 3-μm-thick formalin-fixed, paraffin-embedded sections of the renal tissue samples were deparaffinized and rehydrated. Antigens were retrieved by treatment with high-pressure steam for 10 min. Subsequently, endogenous peroxidase was quenched with 0.3% H_2_O_2_ in the dark for 30 min. The sections were incubated with monoclonal mouse anti-EBV-LMP1 (0.2 μg/ml; DakoCytomation Corporation, Carpinteria, CA, USA) overnight at 4°C. Subsequently, the sections were incubated with rabbit anti-mouse horseradish peroxidase-conjugated IgG (IgG-HRP; Beijing Zhongshan Golden Bridge Biotechnology Co. Ltd., Beijing, China) for 30 min at room temperature. Between the steps, the sections were washed in phosphate-buffered saline (PBS) with three changes. Color was developed with a diaminobenzidine (DAB) kit (Wuhan Boster Biological Technology Ltd., Wuhan, China). Negative control tests were performed by replacing the primary antibody with a non-specific mouse monoclonal antibody (Biolegend, San Diego, CA, USA). Known EBV-positive undifferentiated nasopharyngeal carcinoma (NPC) specimens which were collected from the Department of Pathology (the Affiliated Hospital of Guangdong Medical College) were set as the positive controls. The sections were counterstained with hematoxylin prior to mounting.

### Detection of EBV-encoded RNA 1 (EBER-1) expression using in situ hybridization (ISH)

An ISH for EBER-1 test kit was purchased from Triplex International Biosciences (China) Co., Ltd. (Fuzhou, China). The detection procedures were conducted strictly according to the manufacturer’s instructions, which included the following four steps sequentially: i) Hybrid pre-treatment: 4-μm-thick sections were routinely dewaxed and hydrated, then digested by proteinase K (25 μg/ml) at room temperature for 4.5 min; ii) hybridization: Following washing in distilled water for 1 min, 15–20 μl EBER-1 probe was added to the sections, which were then covered with coverslips (provided in the kit), degenerated at 70°C for 15 min and annealed for 10 min on ice. Subsequently, the slices were incubated at 37°C for 16 h in a humidity chamber containing 30% formamide solution. iii) Hybrid post-processing: The sections were soaked in 48°C PBS for 5 min to remove the coverslips, followed by washing in 48°C PBS for 5 min three times. The sections were incubated with mouse anti-digoxin antibody [Triplex International Biosciences (China) Co., Ltd. (Fuzhou, China)] at 37°C for 2 h in a humidity chamber containing distilled water, then with polymer enhancer solution for 40 min at room temperature, and with polymerized HRP-anti-mouse IgG for 1 h at room temperature. Between steps, the sections were washed in PBS at room temperature for 2 min three times. iv) Coloring and mounting process: Color was developed with DAB solution for 2–10 min and the reaction was stopped according to microscopic evaluation. The sections were counterstained with hematoxylin and then mounted with neutral gum. Negative controls tests were conducted by adding hybridization solution without a probe. Known EBV-positive undifferentiated NPC specimens were used as the positive controls.

### Serum autoantibody determination

Anti-nuclear antibodies (ANA) in the serum of patients with LN were determined using an ELISA kit (Medibiotech Ltd., Tianjin, China). The anti-extractable nuclear antigen (anti-ENA) profiles, including anti-RNP, anti-SSA, anti-SSB, anti-Jo-1, anti-Sm and anti-ds-DNA, were determined using an ENA Profile ELISA kit (Medibiotech Ltd.).

### Statistical analysis

The statistical software SPSS version 15.0 (SPSS. Inc., Chicago, IL, USA) was used to perform the statistical analysis of the data. The equivalence of EBV-LMP1 detection by IHC and EBER-1 detection by ISH was assessed by the McNemar and κ tests. Comparison of the rate of EBV-LMP1 or EBER-1 expression was performed using the χ^2^ test. To investigate the association of kidney-expressed EBV-LMP1/EBER-1 with serum autoantibodies, the patients were divided into renal EBV-expressing and non-expressing groups. The proportions of the patients exhibiting sera autoantibodies were compared between groups using the χ^2^ test.

## Results

### Distribution of EBV markers in renal tissue

**Figure 1:**
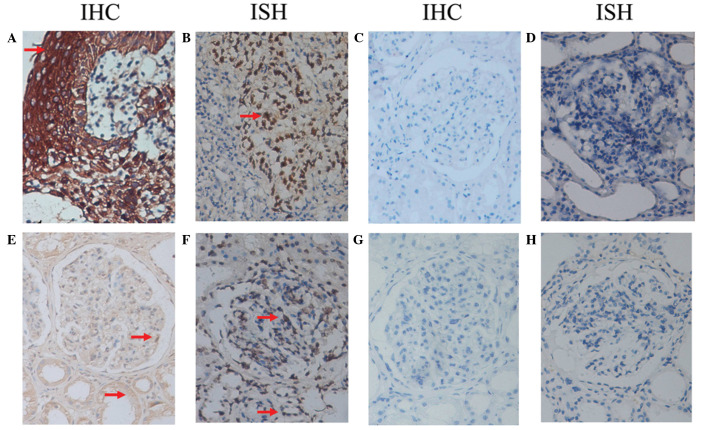
Distribution of EBV-LMP1 and EBER-1 in renal tissue. (A) EBV-LMP1 was strongly expressed in the cytoplasm and (B) EBER-1 was strongly expressed in the nuclei in the undifferentiated NPC specimens. (C and D) Renal tissues that were negative for EBV-LMP1 and EBER-1 expression, respectively. In the renal tissue in which positive expression was observed, (E) EBV-LMP1 was mainly expressed in the cytoplasm of renal tubular epithelial cells and less expressed in the cytoplasm of the podocytes, mesangial cells and endothelial cells of the glomeruli, (F) while EBER-1 was mainly expressed in the nuclei of the renal tubular epithelial cells, podocytes, mesangial cells and endothelial cells. (G) When the primary antibody was replaced with a non-specific mouse monoclonal antibody, EBV-LMP1 expression was not detected in the positively expressing renal tissue. (H) When hybridization solution without a probe was added, EBER-1 was not detected in the positively expressing renal tissue. IHC, immunohistochemistry; ISH, *in situ* hybridization; EBV, Epstein-Barr virus; LMP1, latent membrane protein-1; EBER-1, EBV-encoded RNA 1; NPC, nasopharyngeal carcinoma tissues. Red arrows indicate EBV-LMP1-positive expression in IHC staining and EBER-1 positive expression in ISH staining.

EBV-LMP1 was mainly expressed in the cytoplasm of the renal tubular epithelial cells and was expressed at lower levels in the cytoplasm of the podocytes, mesangial cells and endothelial cells of the glomeruli. EBER-1 was mainly expressed in the nuclei of the renal tubular epithelial cells, podocytes, mesangial cells and endothelial cells. However, as identified by ISH and IHC, there was less positive staining in the renal tissue samples than in the undifferentiated NPC specimens under the same experimental conditions (Fig. 1).

### Agreement of the results of IHC and ISH

Of the total 102 renal tissue samples, 42 cases were identified as expressing EBV-LMP1, while 41 renal tissue samples were identified as expressing EBER-1. A total of 37 renal tissue samples were positive for EBV-LMP1 and EBER-1 while 56 renal tissue samples were negative for both. The McNemar test revealed that there was not a statistically significant difference between the results of the two detection methods (P=1.00) and the κ coefficient was 0.817 (P<0.001). These results showed that there was a high degree of consistency between the two detection methods.

### Positive rates of renal EBER-1 and EBV-LMP1 expression

EBV-LMP1 was identified in 34 (58.6%) of the renal tissue samples from patients with LN, while there was only one (14.3%) renal tissue sample in the normal group and seven (18.9%) renal tissue samples in the minimal change nephropathy group in which the expression of EBV-LMP1 was identified. The positive rate of renal EBV-LMP1 expression in the LN group was significantly higher than those of the normal and minimal change nephropathy groups (P<0.001). EBER-1 was identified in 35 (60.3%) of the renal tissue samples with LN and six (16.2%) renal tissue samples with minimal change nephropathy, while no normal renal tissue samples (0.0%) were identified to express EBER-1. The positive rate of renal EBER-1 expression of the LN group was significantly higher than those of the normal and minimal change nephropathy groups (P<0.001), while no significant difference was identified between the normal and minimal change nephropathy groups (P>0.05; Table I).

### Renal expression of EBV-LMP1 and EBER-1 in patients with LN and different clinical statuses

The positive rate of EBV-LMP1 and EBER-1 expression in the renal tissue samples with LN was not observed to be statistically different between the initial onset (non-treated) and recurrent patients (immunosuppressant-treated) and between the patients with and without concurrent infection (P>0.05; Table II).

### Positive rates of renal EBV-LMP1 and EBER-1 expression in patients with LN of different age ranges

When the LN patients were divided according to age (0–19, 20–39 and ≥40 years), no significant differences in the positive rates of renal EBV-LMP1 and EBER-1 expression were identified among the three age groups (Table III).

### Association of renal EBV-LMP1/EBER-1 expression with autoantibody production in patients with LN

The positive rate of serum anti-Sm in the LN patients was significantly higher in the renal EBV-expressing group than in the non-expressing group (P<0.05), while the positive rates of serum ANA, anti-RNP, anti-SSA, anti-SSB, anti-Jo-1 and anti-ds-DNA were not found to be significantly different between the two groups (P>0.05) (Table IV).

## Discussion

EBV infection and NPC are highly prevalent in southern China (15). Primary EBV infection usually occurs in childhood, and is asymptomatic and latently infectious. However, if primary infection occurs in adolescence or adulthood, it may result in infectious mononucleosis syndrome. Primary infection may contribute to viral persistence in the human body, manifesting with an asymptomatic latent infection status (16–18).

The detection methods for human EBV infection include detecting EBER-1 expression or EBV DNA by ISH, detecting EBV DNA by Southern blotting, detecting the expression of a variety of EBV antigens by IHC, detecting the expression of a variety of EBV antigens by serological methods, and detecting viral particles by electron microscopy. EBER-1 is a small RNA molecule without a poly A tail that is not translated into proteins. Copy numbers of EBER-1 are very high and reach 10^6^ copies in a single host cell nucleus, and EBER-1 is currently the most abundant viral RNA during EBV latent infection. Detection of EBER-1 by ISH is considered as the gold standard for the identification of EBV as it precisely identifies the sites of expression and is highly sensitive and specific; however, ISH is expensive (19). EBV-LMP1 is a rich protein product of EBV. Due to relatively inexpensive detecting reagents, detection of EBV-LMP1 by IHC is usually employed to screen for EBV infection in clinical practice (20). The results of the present study showed that the two aforementioned detection methods had strong agreement, which indicated that the results were reliable.

In the present study, it was observed that the EBV-LMP1 expression intensity and EBER-1 hybridization in the renal tissue samples were weaker than those in the NPC tissues (the positive controls) under the same experimental conditions, which suggested that the number of copies of EBV in the renal tissues was lower than that in the NPC tissues. Additionally, EBV-LMP1, a transmembrane protein, would theoretically be only distributed in the cytoplasm and membrane, which was confirmed in the positive control specimens. Unexpectedly, it was also expressed in some of the nuclei of podocytes, glomerular mesangial cells, glomerular endothelial cells and renal tubular epithelial cells, which requires further investigation.

It was found that the EBV positive rate in the renal tissue samples with LN was significantly higher than those of the renal tissue samples with minimal change nephropathy and non-nephropathy, suggesting that EBV infection may play a role in the pathogenesis of LN. Two hypotheses may explain these results: i) individuals with LN genetic susceptibility may easily develop LN following EBV infection and ii) intrinsic immune disorder and immunosuppressant treatment in patients with LN may lead to EBV infection vulnerability. In order to screen out the latter possibility, the positive rate of the expression of two virus markers was compared between initial onset (non-treated) and relapse (immunosuppressive agent-treated) patients, patients with and without complicating clinical infection, as well as among patients in different age ranges. The results indicated that the expression of the renal EBV markers (represented by the positive rate) was not influenced by immunosuppressive agent treatment, concurrent infection (the majority of cases being respiratory tract infection) or age. These results suggest that EBV infection possibly occurs prior to the onset of LN. Certain susceptible individuals may develop LN due to the inductive effect of EBV infection.

To further confirm our hypotheses, the association between renal EBV infection and autoantibody production was analyzed in the LN patients. It was found that the positive rate of anti-Sm was higher in the group positive for the renal EBV marker than in the group that was negative for it. This result suggested that anti-Sm production may be associated with EBV infection. Anti-Sm is highly specific and the detection rate is ~20–25% in SLE patients (21). EBNA-1 is an important EBV nuclear antigen, which contains a region of PPPGRRP that has been considered to be highly homologous with the Sm antigen region PPPGMRPP (22). Poole *et al* (23) reported that rabbits and rats immunized with PPPGRRP or PPPGMRPP peptide fragments presented lupus-like autoimmunity by producing similar autoantibodies. These results suggest that EBV infection may result in SLE due to molecular mimicry.

In conclusion, the present study suggests that renal EBV infection may be involved in the pathogenesis of LN, and the mechanism is likely to be associated with the induction of autoantibody production.

## Figures and Tables

**Table I:** Positive rates of EBER-1 and EBV-LMP1 expression in the renal tissues of patients with LN, MCN and non-nephropathy.

	EBER-1			EBV-LMP1		
		
Condition	Negative	Positive	Positive rate (%)	Negative	Positive	Positive rate (%)
Normal	7	0	0.0	6	1	14.3
MCN	31	6	16.2	30	7	18.9
LN	23	35	60.3^a^	24	34	58.6^a^

a P<0.01, compared with MCN and normal.

EBER-1, EBV-encoded RNA 1; EBV, Epstein-Barr virus; LMP1, latent membrane protein-1; LN, lupus nephritis; MCN, minimal change nephropathy.

**Table II:** Positive rates of renal EBER-1 and EBV-LMP1 expression in patients with LN and different clinical statuses.

	EBER-1			EBV-LMP1		
		
Clinical status	Negative	Positive	Positive rate (%)	Negative	Positive	Positive rate (%)
Disease course						
Initial onset	9	13	59.1	9	13	59.1
Relapse	14	22	61.1	15	21	58.3
Concurrent infection						
Without	9	17	65.4	11	15	57.7
With	14	18	56.3	13	19	59.4

EBER-1, EBV-encoded RNA 1; EBV, Epstein-Barr virus; LMP1, latent membrane protein-1; LN, lupus nephritis.

**Table III:** Positive rates of renal EBER-1 and EBV-LMP1 expression in patients with LN of different age ranges.

	EBER-1			EBV-LMP1		
		
Age range	Negative	Positive	Positive rate (%)	Negative	Positive	Positive rate (%)
0–19 years	4	7	63.6	4	7	63.6
20–39 years	14	19	57.6	15	18	54.6
≥40 years	5	9	64.3	1	13	64.3

EBER-1, EBV-encoded RNA 1; EBV, Epstein-Barr virus; LMP1, latent membrane protein-1; LN, lupus nephritis.

**Table IV:** Positive rates of serum autoantibodies between patients with and without renal EBER-1/EBV-LMP1 expression.

	Positive rate (%)						
	
EBV markers	ANA	anti-Sm	anti-RNP	anti-SSA	anti-SSB	anti-Jo-1	anti ds-DNA
EBER-1							
Negative	73.9 (17/23)	8.7 (2/23)	13.0 (3/23)	13.0 (3/23)	4.3 (1/23)	0.0 (0/23)	73.9 (17/23)
Positive	68.6 (24/35)	34.3 (12/35)^a^	14.3 (5/35)	8.6 (3/35)	2.9 (1/35)	2.9 (1/35)	77.1 (27/35)
LMP1							
Negative	79.2 (19/24)	8.3 (2/24)	16.7 (4/24)	20.8 (5/24)	8.3 (2/24)	0.0 (0/24)	70.8 (17/24)
Positive	64.7 (22/34)	35.3 (12/34)^a^	11.8 (4/34)	2.9 (1/34)	0.0 (0/34)	2.9 (1/34)	70.6 (24/34)

a P<0.05, compared with negative expression group.

EBER-1, EBV-encoded RNA 1; EBV, Epstein-Barr virus; LMP1, latent membrane protein-1; ANA, anti-nuclear antibody.
